# Visual Appearance of Nanocrystal-Based Luminescent Solar Concentrators

**DOI:** 10.3390/ma12060885

**Published:** 2019-03-16

**Authors:** Panagiotis Moraitis, Gijs van Leeuwen, Wilfried van Sark

**Affiliations:** Copernicus Institute, Utrecht University, Utrecht 3584 CB, The Netherlands; g.e.vanleeuwen2@students.uu.nl (G.v.L.); W.G.J.H.M.vanSark@uu.nl (W.v.S.)

**Keywords:** luminescent solar concentrators, nanoparticles, building integrated photovoltaics, color rendering index, correlated color temperature, Monte Carlo, quantum dots

## Abstract

The luminescent solar concentrator (LSC) is a promising concept for the integration of photovoltaic (PV) generators into the building envelope. Having the form of semitransparent plates, LSCs offer a high degree of flexibility and can be used as windows or facades, as part of the of building-integrated photovoltaic (BIPV) industry. Existing performance characterizations of LSC devices focus almost exclusively on electric power generation. However, when used as window components, the transmitted spectrum can alter the color, potentially affecting the visual comfort of the occupants by altering the properties of the sunlight. In this study, eight different state-of-the-art nanocrystals are evaluated as potential candidates for LSC window luminophores, using Monte Carlo simulations. The transparency of each LSC window varies between 90% and 50%, and the color-rendering properties are assessed with respect to the color rendering index (CRI) and the correlated color temperature (CCT). It is found that luminophores with a wide absorption bandwidth in the visible spectrum can maintain a high CRI value (above 85) and CCT values close to the Planckian locus, even for high luminophore concentrations. In contrast, luminophores that only absorb partly in the visible spectrum suffer from color distortion, a situation characterized by low CCT and CRI values, even at high transmittance.

## 1. Introduction

In the context of sustainable development, the building sector has attracted increasingly worldwide attention as it consumes more than 30% of the energy produced globally and is responsible for about 40% of both direct and indirect ${\mathrm{CO}}_{2}$ emissions [1]. Technological advances towards sustainable buildings have led to an annual reduction of 1.3% in energy intensity per unit of floor area; however, as growth overwhelms the improvements, the global electricity demand for building is growing by a rate of 2.5% a year [2]. As a direct response, the European Commission, from 2010, has released directives for newly constructed buildings to have a near-zero net energy balance [3].

Power generation directly from solar radiation is a major step towards sustainable electricity production, providing clean energy without the environmental impact caused by excessive carbon emissions. Solar photovoltaic (PV) technology [4] is growing rapidly and, by the end of 2017, approximately 400 GW of cumulative installed capacity generated more than 460 TWh of electricity, a figure that represents around 2% of the global power output [5]. The leader of this solar revolution is undoubtedly the silicon PV module. With commercial efficiency values that exceed 20% and constantly decreasing manufacturing and installation costs, the silicon solar cell is the workhorse of energy production in both multi-megawatt solar farms and small domestic installations [6]. Conventional solar panels are considered to be a mainstream and cost-effective solution for both public and private buildings but, so far, they have not managed to integrate into densely-populated urban areas [7].

Luminescent solar concentrators (LSCs) are light-harvesting devices that have the potential to revolutionize future urban architecture, by introducing an efficient [8,9], low cost [10] building-integrated photovoltaic (BIPV) solution, in the form of transparent or semitransparent solar energy generators that could be integrated into the building envelope as windows or facades. LSCs, unlike any other optic-based concentration device, do not require any type of sun tracking mechanism and offer the capability to absorb both direct and diffuse irradiation [11]. Typically, a LSC consists of a plastic or glass sheet containing highly luminescent molecules. Under solar illumination, the luminescent molecules (or luminophores) absorb the incoming photons, which are re-emitted at longer wavelengths andm through total internal reflection, are guided towards the edge of the sheet. The concentrated emission is collected by PV cells attached to the side of the waveguide, where photon energy is converted into useful electricity [8]. Therefore, LSCs have the potential to reduce the overall cost of the generated electricity by increasing the incident radiation flux, while, at the same time, reducing the amount of PV material needed. They are also promising BIPV candidates, as the waveguide, which is commonly the only visible part of the configuration, has a tremendous degree of flexibility in terms of shape, color, and transparency level [12].

Undoubtedly, the most critical element for the operation of an LSC device is the selected luminophore, as the absorption and emission spectrum directly dictates the amount of photons that will be absorbed and, consequently, re-emitted. This process determines the amount of the concentrated energy on the edge of the LSC and, therefore, the final power output of the device [13]. Luminescent species also shape the spectrum of the light that is transmitted through the LSC and, therefore, are also responsible for coloring of the device and the level of transparency [14]. A number of niche applications of LSCs that have been presented so far, such as greenhouse glass [15], sound barriers [16], decorative elements [17] and solar tiles [18], were hardly affected by the strong coloring imparted by the luminophores used.

However, in the context of an LSC as a window component, the distribution of the transmitted spectrum can affect the color quality of the natural light in the interior of a building [19,20]. Although visual comfort parameters, such as color neutrality and color rendering, are common in architectural glass and glazing applications [21] they are not usually taken into account in LSC evaluation studies. Lighting conditions contribute to well-being [22], and are also related to human performance and health—with recent studies showing that insufficient lighting might disrupt standard human rhythms and affect physical, physiological, and psychological behaviors [23,24]. Other studies have shown that occupants have a clear preference of certain lighting levels over others [25]. The results from one study, focused particularly on the effects of red-coloured LSCs, suggested that any coverage of the window over 25% has a negative effect on the experience of the occupants, with a 50% coverage being the maximum acceptable level [26].

The objective of this study is to evaluate eight state-of-the-art luminophores as candidates for LSC window applications, using ray-tracing Monte Carlo simulations. The LSC plates will have a varying range of transparency, from 90% to 50% for each luminophore, and will be assessed with respect to different color quality criteria and performance indicators. First, we discuss colorimetric theory to give an overview on how human vision perceives colors and how they can be evaluated. Then, we discuss the types of the luminophores we selected and their properties, followed by an analysis of how Monte Carlo simulations are used in this context.

## 2. Colorimetry

**Figure 1:**
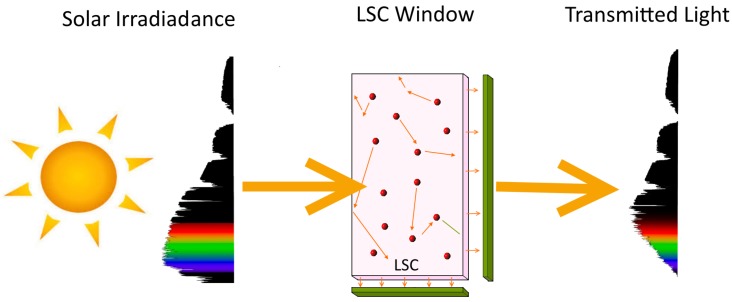
Schematic representation of solar radiation passing through a luminescent solar concentrator (LSC) window with 2 photovoltaic (PV) cells attached to the sides. A portion of the short wavelength has been absorbed by the LSC, changing the spectral power density (SPD) of the transmitted light.

The idea of using LSCs as parts of the building envelope, and specifically as windows, has been thoroughly examined in recent years [8,9]. The concept is simple: Part of the incident daylight will be captured by the luminophores to be waveguided and converted into electricity, while the rest will pass through to illuminate the interior. This process will primarily result in a lower transmitted power *P*, compared to the incident power $P_{0}$ and, additionally, will affect the shape of the transmitted spectral power distribution (SPD); see Figure 1. Therefore, depending on the optical properties of the luminophores, even if two LSC devices exhibit similar luminous transmittance values, they may have quite different spectral transmittance. This may result in a different appearance of objects in the building’s interior.

What the human eye perceives as daylight is part of the solar radiation in the visible range, from 380 nm to 780 nm. The SPD of this relatively small part of the solar spectrum can affect visual comfort and the color perception in a room. Even without any human intervention, the SPD of natural daylight varies depending on the time of the day, weather conditions, and geographical location. The variation of natural daylight is usually described by using the correlated color temperature (CCT), which is measured in Kelvin (K), and it is the temperature of a black-body radiator with the closest chromaticity to the light source [27]. Under clear-sky conditions during a regular day, the CCT of natural daylight can vary from 2000 K in the early morning to 20,000 K in the late afternoon [28]. Low CCT values imply a warmer color that looks yellowish to human eyes, such as candle light with a CCT of 1500 K or an incandescent lamp with 3000 K. On the other hand, higher CCT values indicate a more bluish color. The CCT of the average daylight at noon is 6500 K.

It is not possible to measure color directly, as it is always based on comparison [27]. Moreover, in order to see colors, light is necessary; under different illuminants, colors may appear different. Therefore, a reference system is necessary. The same applies for the color quality of a light source, a reference source is needed for comparison. When an object of any color is observed under different light sources with different SPDs, the perception of the color slightly changes for the viewer. This conscious or subconscious comparison of the color appearance of objects, with the color appearance under a reference or a standard illuminant, is defined as color rendering [29]. The Commission Internationale de l’Eclairage (CIE), a non-profit organization considered to be the authority on the science of light and color, introduced the standard illuminant as a light source with a specific SPD. Standard illuminants include common light sources such as average daylight, as well as incandescent and fluorescent lamps. CIE has also standardized the average midday light in Western Europe as the CIE Standard Illuminant D65, which is to be used in all colorimetric calculations requiring a representative daylight.

To quantify the ability of a light source to render various colors, the Color Rendering Index (CRI) is used. This method uses the light from a test source, such as the light coming from an LSC window, and measures how the color of reflected light appears, compared to eight basic and six additional Munsell color samples; see Table 1 [27]. These color samples have been measured under a reference light source, and the difference between the color under the test light and the color under the reference light is used to calculate the CRI. The CRI varies from 0 to 100 and the higher the value, the better the color rendering ability of the test source.

Color rendering first became an issue in the twentieth century, with the introduction of commercial fluorescent lamps. These newly-discovered light sources could provide a wide range of colors and, therefore, a wide range of possible SPDs. For comparison, white fluorescent tubes have a CRI value around 60, standard LED lights score around 80, and only incandescent lamps score 100—which are, effectively, almost blackbody radiators. Depending on the application, the appropriate CRI value is selected; for example, highway lighting is usually below 70, while an office or work environment requires a minimum of 80. In architectural daylight concepts, where CRI defines the spectral transmissive quality of windows, values above 90 offer a good level of color neutrality, and values above 95 are considered as excellent [28].

The main criticism of CRI is focused on the fact that it is not possible to quantify with a single value, given the way that colors can be affected by a change of illuminant. This single-valued color fidelity approach fails to properly assess the color rendition properties of light sources with narrow-band SPDs, such as LED lighting [30]. Furthermore, by calculating the arithmetic mean of the errors on the test colors, it is possible to diminish the contribution of any single large deviation. Additionally, since all errors are weighted equally, the indicator can be considered as non-perceptual, since human vision inherently has a preference for certain colors. However, despite these criticisms, the CRI still remains the most widely-accepted color rendering indicator and, therefore, will also be used in the current study.

## 3. Luminophores

The most fundamental component of an LSC device is undoubtedly the luminophores, as they are the driving force for solar concentration. By designing LSCs as window elements, the role of the luminophores becomes almost counter-intuitive. On one hand, they should allow for maximum light transmittance in the visible region, in order to provide the same comfort level as a conventional window, but, on the other hand, they should achieve maximum absorption and re-emission of light within the waveguide.

This combined need of maximum concentration and maximum efficiency is a demanding process, where every photon counts. Therefore, an effective luminophore should be characterized by a series of properties. Primarily, a broad absorption spectrum combined with high absorption efficiency in this range is necessary, to ensure that incoming photons will be captured within the limited width of a glass window. Then, a high luminescent quantum efficiency ($\eta_{LQE}$) is also required, in order for the absorbed photons to be re-emitted effectively. However, the greatest challenge in luminophore development is the elimination of re-absorption. The small energy separation between the emission band and the absorption peak (the Stokes shift), leads luminophore-emitted photons to be re-absorbed by subsequent luminophores as they travel through the waveguide. The re-absorption process is not necessarily a loss by itself, but it becomes a loss through the non-unity quantum yield efficiency or the re-emission in a direction within the escape cone. Finally, for the efficient conversion of the guided luminescence into electricity, the energy of the emitted photons should have a good match with the PV cell attached to the sides of the LSC device.

While these conditions are necessary to maximize the solar-harvesting capabilities of a given luminophore, the creation of a luminophore that will also be suitable for LSC window devices creates additional requirements. The emergence of a new BIPV element that enters a mass-production phase would require high volumes of raw materials. Materials with relatively low abundance could cause supply limitations and price fluctuations. Additionally, luminphores that consist of toxic elements or heavy metals could be restricted by safety regulations. Therefore, synthesizing a suitable luminophore that could fulfil all the prerequisites has been proven to be a difficult task. The luminophores studied in this paper each use different approaches to tackle one or more of the aforementioned issues.

**Figure 2:**
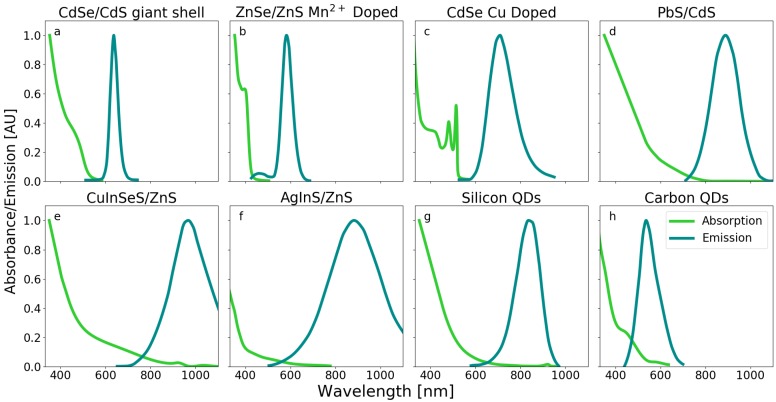
Absorption and emission spectra of eight different luminophore candidates for LSC windows. Data acquired from (**a**) [32] (**b**) [35] (**c**) [36] (**d**) [37] (**e**) [38] (**f**) [39] (**g**) [40] (**h**) [41].

Colloidal chemistry exhibits some very attractive features for the development of Quantum Dots (QDs) as luminophores [31]. Solubility, cheap fabrication, and processing of the nanocrystals are crucial factors for scaling-up production, while tunable properties could help eradicate reabsorption and increase performance. For this purpose, the realization of core/shell hetero-nanocrystals is a promising technique for Stokes-shift engineering. This principle is also followed in the development of thick-shell CdSe/CdS QDs [32], where the wider energy gap material of the shell acts like a light harvesting antenna, while, at the same time, offering protection and isolation to the core, which primarily dominates the emission [33]. By growing a thick CdS shell around a CdSe core, a large Stokes shift is achieved, with peak emission at 640 nm and tail absorption up to 600 nm (see Figure 2a). At the same time, $\eta_{LQE}$ is relatively high, reaching 45%.

However, as the size of the shell increases, light scattering becomes a non-negligible loss [34]. Therefore, instead of growing a thick shell to tackle reabsorption, an alternative approach is to introduce a luminescence activator in the QD, to allow new, optically-forbidden transitions within the bandgap of the semiconducting materials. In the case of ${\mathrm{Mn}}^{2+}$-doped ZnSe/ZnS QDs [35], the semiconductor host provides absorption in the UV range of the solar spectrum, which has a peak at 400 nm, while the emission is dominated by the Mn impurity at 590 nm (see Figure 2b) with an $\eta_{LQE}$ of 50%. By absorbing only at the UV part of the spectrum, Mn-doped ZnSe/ZnS QDs has the benefit of letting the visible light pass through, which is an ideal feature for a window application. However, only 10% of the incoming irradiation can be captured with this type of luminophore. An alternative approach is to use the broader absorption of CdSe, which expands into the visible part with a sharp absorption peak at 465 nm (see Figure 2c), combined with the redshifted emission of ${\mathrm{Cu}}^{+}$ impurities, resulting in a Cu-doped system, with a peak wavelength at 705 nm. The result is a colloidal quantum well (CQW) structure, with $\eta_{LQE}=70\%$ in a PLMA (polylaurylmethacrylate) matrix; however, this contains heavy metal elements [36].

Even with minimum reabsorption, high efficiency values require a broad absorption spectrum that can capture a larger fraction of solar irradiation. Doped QDs and core/shell structures, however, have a limited spectral range (less than 500 nm) that covers only the UV and visible parts of the spectrum. By using lead chalcogenides, such as PbS with a shell of CdS, the absorption cross-section expands to the near-infrared (NIR) (Figure 2d), while the quantum efficiency is maintained at 50% [37]. Even though these types of QDs contain both Pb and Cd, which are known to be toxic, when encapsulated in an LSC device they are effectively sealed from the external environment [42]. Interestingly, an alternative use of copper is as a compound in non-toxic ternary I–III–${\mathrm{IV}}_{2}$ semiconductors [43,44]. The CuInSeS QDs coated with ZnS have an emission peak at 960 nm, which is optimal for coupling with Si PV cells and a broad absorption range that exhibits a weak shoulder at 640 nm, covering the UV and visible wavelengths (Figure 2e) [38]. The wider-gap ZnS shell protects the core, maintaining a relativity high $\eta_{LQE}$ at 40% [45]. Similarly, heavy metal-free AgIn$S_{2}$/ZnS exhibit NIR emission at 900 nm, but with a slightly narrower absorption range and with a lower $\eta_{LQE}$ value at 30% (Figure 2f) [39].

A completely different material design focuses on one of the most abundant elements in the Earth’s crust, Silicon. Although it is known as an indirect-bandgap semiconductor, upon quantum confinement it becomes strongly luminescent [46]. Si QDs exhibit a smooth absorption profile, as typical in indirect-bandgap semiconductors, and an emission peak at 830 nm (Figure 2g) [40]. With limited scattering losses and $\eta_{LQE}=45\%$, they can allow for large-scale industrial fabrication of reliable LSC emitters. A non toxic, low cost, and scalable solution can be found in carbon QDs, which additionally offer excellent air stability and, therefore, can be stored in ambient conditions [47]. Carbon QDs have a broad absorption spectrum, in the range of 300–600 nm, with a peak at 350 nm, and the luminescence has a maximum at 550 nm (Figure 2h), with $\eta_{LQE}=40\%$ [41]. The emission peak, quantum yield, and the full width at half maximum of the luminophores studied in this research can be seen in Table 2.

## 4. Method

### 4.1. Overview

**Figure 3:**
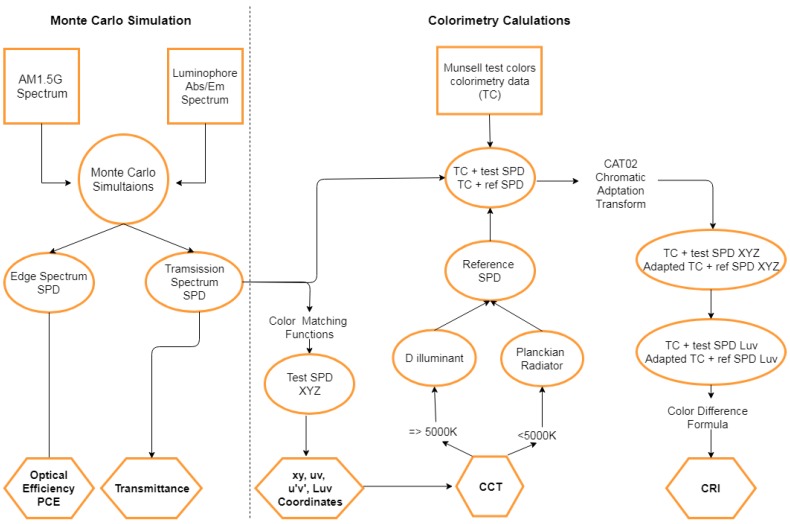
Structure of the methodology. Inputs are placed in a rectangular shape, the ellipses represent intermediate results, and the hexagons represent the end results.

To calculate the efficiency and the colorimetric performance of LSC windows containing the luminophores, as described in Section 3, the method presented in Figure 3 will be followed. The absorption and the emission spectrum of the luminophores will be used in Monte Carlo simulations to obtain the edge and transmission SPDs (Figure 3, left side) and, from the latter, the CCT and CRI will be calculated (Figure 3, right side). This will be thoroughly discussed in Section 4.2 and Section 4.3.

### 4.2. Monte-Carlo Simulations

A Monte-Carlo or ray tracing simulation is a common technique in LSC research, as they allow for quick and accurate evaluation of different properties, such as device size, geometry, luminophore type, and concentration. While it is mainly used to calculate the efficiency of a device, useful outcomes can be obtained by studying photon paths and properties. The basic principle is to compute the path of a single photon for a given set of initial conditions. These conditions include the position and the spectrum (S(λ)) of the light source, the geometry and the refractive index of the LSC device (*n*), and the properties of the luminophores: Absorption spectrum (A(λ)), emission spectrum (E(λ)), and the quantum yield ($\eta_{LQE}$).

The simulation software [48] generates photons at the light source, with emission wavelength calculated from the probability density integral equation for a randomly generated number ξ∈ [0,1].

(1) $$\frac{1}{K}\int_{0}^{\lambda_{em}}S\left(\lambda \right)d\lambda =\xi ,$$

where *K* is a normalization constant, calculated as:

(2) $$K=\int_{0}^{\infty }S\left(\lambda \right)d\lambda .$$

Once a wavelength has been assigned to the photon, it is then emitted from the source. The source, unless it is stated otherwise, is located above the simulated LSC device and emits photons perpendicular to the upper surface of the device. It is also possible to place the source with a specific angle, or to emit photons from a hemisphere that has the LSC device in the center, in order to simulate diffuse irradiation. Upon interaction with any material that has a different refractive index, the probabilities for reflection and refraction are calculated according to the Fresnel equations for unpolarized light, accounting for the incident angle.

Once a photon is within an absorbing medium, the path length *l* is calculated using the Lambert–Beer law:

(3) $$A\left(\lambda \right)=1-10^{-\epsilon (\lambda )cl},$$

where ϵ(λ) is the absorption coefficient of the absorbing material, with respect to the wavelength λ of each individual photon, and c is the concentration of the absorbing material. The path length *l* is calculated by replacing the absorption probability by a random number, which is generated from the uniform distribution within [0,1]. This path will determine the point where the photon will be absorbed. In the case where the path is longer than the physical dimensions of the LSC device, then it will go through the Fresnel equations again to determine whether there will be refraction, and the photon will be lost, or it will continue within the medium due to internal reflection.

In the case of photon absorption by the absorbing material, the luminescence quantum efficiency will determine whether the photon will be re-emitted or be lost. If the photon is re-emitted, then two parameters should be determined: The first one is the direction of the new photon and the second is the wavelength. The direction is calculated by generating a random vector on a unit sphere centered on the point of absorption, based on the principle of isotropic emission. The new wavelength is calculated in similar way as in Equation (1), using the emission spectrum E(λ) instead of the source spectrum S(λ), and with the limitation $\lambda_{2}>\lambda_{1}$, as the new wavelength can only be longer after re-emission.

(4) $$\frac{1}{K}\int_{\lambda_{abs}}^{\lambda_{em}}E\left(\lambda \right)d\lambda =\xi ,$$

where *K* is a normalization constant, calculated as:

(5) $$K=\int_{\lambda_{abs}}^{\infty }S\left(\lambda \right)d\lambda .$$

This process will terminate either if the photon is out of the device, or is not re-emitted after an absorption event. Then, a new photon will be generated from the light source. All the information generated during the photon path such as wavelength, position, direction, absorption events, and surface interactions are stored in a database. When the ray tracing is complete, the final photon flux collected on the edges and behind the window can be collected. From the latter, the spectrum of the transmitted light can be calculated.

The edge spectrum is necessary for the calculation of optical efficiency ($\eta_{opt}$), which is the ratio of the radiative power on the edge of the LSC ($L_{edge}$) to the radiative power incident on the top of the device ($L_{in}$):

(6) $$\eta_{opt}=\frac{L_{edge}}{L_{in}}.$$

**Figure 4:**
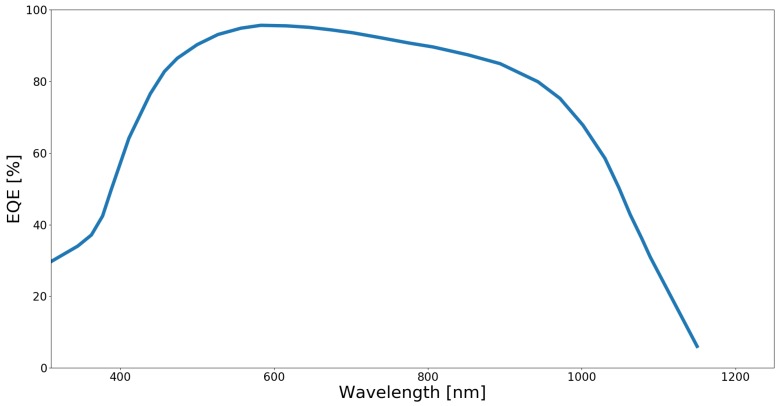
External quantum efficiency (EQE) of the Silicon solar cell [49].

The data that are collected from a ray tracing simulation can be further utilized, for the simulation of the PV elements that are attached to the side of the LSC device. The short circuit current ($J_{sc}$) can be obtained by calculating the amount of free carriers as the integral value of the photon flux (ϕ(λ)), with respect to the external quantum efficiency (EQE) of the solar cell [4]:

(7) $$J_{sc}=q\int_{\lambda_{1}}^{\lambda_{2}}\phi \left(\lambda \right)EQE\left(\lambda \right)d\lambda ,$$

where $\lambda_{1}$ and $\lambda_{2}$ correspond to the minimum and the maximum wavelengths, respectively, of the AM1.5G spectrum used for the simulation. It is common practice in ray tracing simulations to truncate the parts of the solar spectrum that do not interact either with the luminophores or the solar cell, in order to make better use of the available computational resources and decrease the simulation time. In this case, the minimum wavelength was set to 200 nm and the maximum to 1200 nm. For the calculation of the short circuit current, the incident AM1.5G full spectrum (200–4000 nm) was normalized to 1000 W/m${}^{2}$. The EQE of the silicon solar cell used for the simulations is given, in Figure 4, as a function of the photon wavelength. Then, $V_{\mathrm{oc}}$ can be calculated from the following formula:

(8) $$V_{\mathrm{oc}}=\frac{k_{B}T}{q}ln\left(\frac{J_{SC}}{J_{S}}+1\right),$$

where $J_{s}$ is the diode saturation current density, $k_{B}$ is the Boltzmann constant, *T* is the temperature (K), and *q* is the elementary charge unit [4]. The power conversion efficiency $\eta_{PCE}$ of the simulated solar cell can be obtained from:

(9) $$\eta_{PCE}=\frac{V_{\mathrm{oc}}J_{SC}FF}{P_{in}},$$

with FF being the fill factor, as given by the cell manufacturer.

### 4.3. Colorimetric Performance

**Figure 5:**
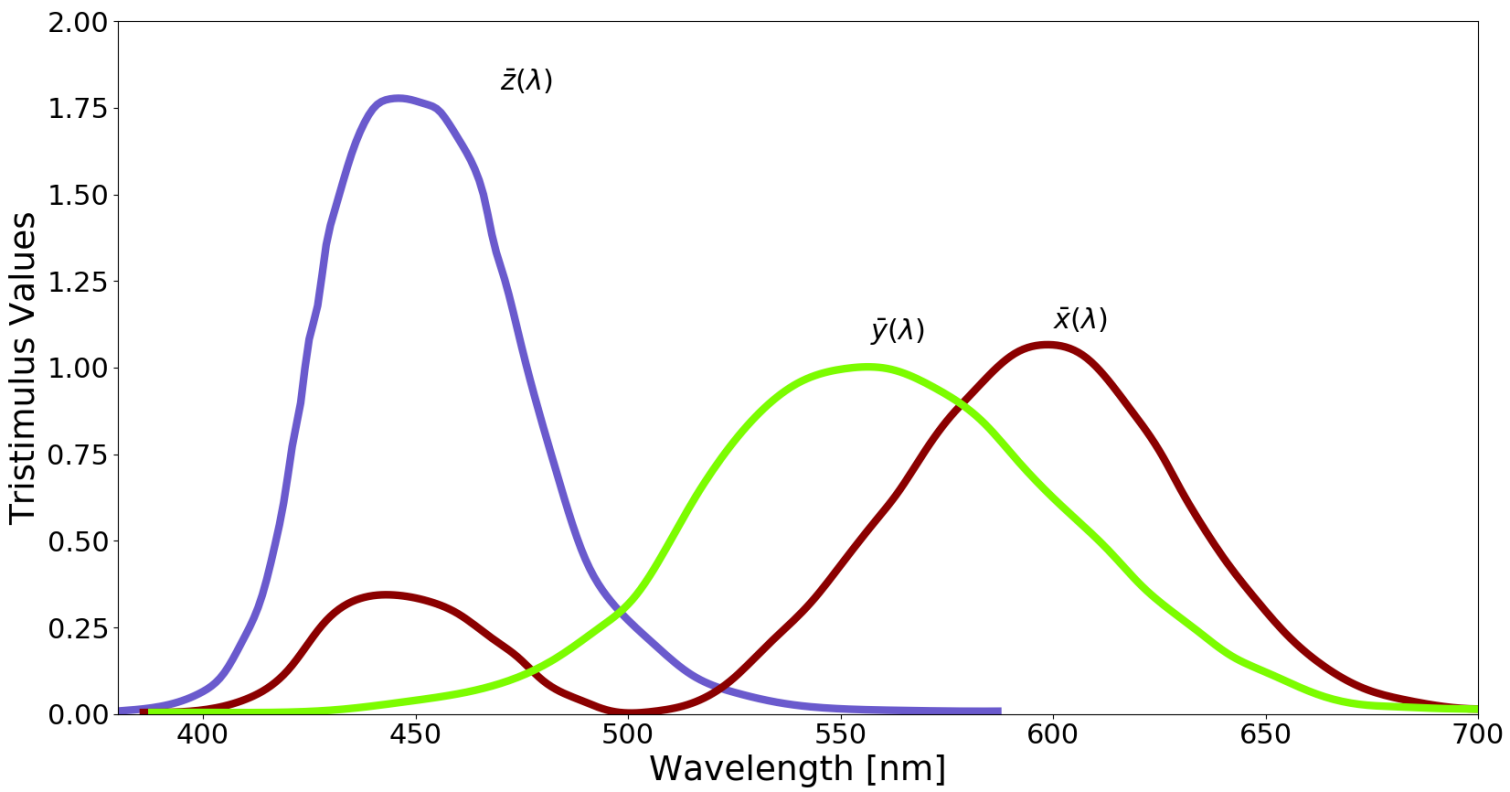
Spectral response of color matching functions $\bar{x}\left(\lambda \right),\bar{y}\left(\lambda \right),$ and $\bar{z}\left(\lambda \right)$ [27].

The tristimulus values (X,Y,Z) are the three color perception values of human vision and they are the most basic quantities in colorimetry. They were introduced in 1931 by the CIE to replace the well known R,G,B (red, green, blue) system, in order to become more practical. X,Y,Z can be computed from the measured SPD and the color matching functions $\bar{x}\left(\lambda \right)$, $\bar{y}\left(\lambda \right)$, $\bar{z}\left(\lambda \right)$, which represent the response of the human eye to certain wavelengths, also known as the photopic curve (Figure 5). The X, Y, and Z tristimulus values are given by the following formulas [50]:

(10) $$X=K\int_{vis}P\left(\lambda \right)\bar{x}\left(\lambda \right)d\lambda ,$$

(11) $$Y=K\int_{vis}P\left(\lambda \right)\bar{y}\left(\lambda \right)d\lambda ,$$

(12) $$Z=K\int_{vis}P\left(\lambda \right)\bar{z}\left(\lambda \right)d\lambda ,$$

where the value P(λ) represents the radiant power at each wavelength interval and the values $\bar{x}\left(\lambda \right)$, $\bar{y}\left(\lambda \right)$, and $\bar{z}\left(\lambda \right)$ represent the values of the three color-matching functions for each wavelength interval. The value K = 683 lm/W is a constant.

The chromaticity coordinates are the ratio of each of the tristimulus values, X,Y, and Z, divided by their sum. As the sum of the chromaticity coordinates is equal to 1, only two of them are necessary to describe a color. They are used to visualize the color of the given stimulus in a 2D chromaticity diagram [51]:

(13) $$x=\frac{X}{X+Y+Z},$$

(14) $$y=\frac{Y}{X+Y+Z}.$$

**Figure 6:**
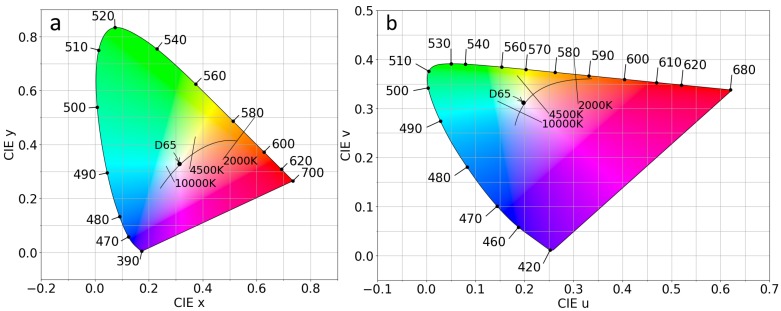
(**a**) The CIE 1931 chromaticity diagram with chromaticity coordinate values (x,y) of the D65 illuminant (average sunlight) and the Planckian locus. (**b**) CIE 1960 Uniform Color Space (UCS). chromaticity diagram with chromaticity coordinate values (u,v) of the D65 illuminant and the Planckian locus. The extended lines represent isotherms.

While the xy chromaticity diagram, as depicted in Figure 6, is very commonly applied, it suffers from a serious downside: The distribution of colors is very non-uniform [27]. This is better illustrated when trying to depict a perceptual color difference of the same magnitude between different colors. Ideally the identical color differences should have been depicted by lines with the same length. Although there is no chromaticity diagram that can totally solve this problem, the development of the $u^{\prime }v^{\prime }$ chromaticity diagram created a more uniform color space. The xy coordinates can be easily translated to $u^{\prime }v^{\prime }$ coordinates, using the following equations [51].

(15) $$u^{\prime }=\frac{4x}{-2x+12y+3},$$

(16) $$v^{\prime }=\frac{9y}{-2x+12y+3}.$$

The CCT can be determined by plotting the test stimulus on a uv chromaticity diagram and taking the temperature of the point on the locus of Planckian radiators that is closest to the test illuminant. The locus of Planckian radiators is a path on the chromaticity diagram that is taken by a black body radiator as its temperature changes. For modelling the determination of the CCT, several methods are available, and the method devised by Robertson in 1968 is found to be most accurate. If the CCT is under 10,000 K, which is usually the case, the error of the method is close to zero. The CCT of a stimulus can only be considered valid if the chromaticities are close enough to the Planckian locus [27].

Chromaticity diagrams are used in many different applications but, as follows from Equations (13)–(16), they only show the proportions of tristimulus values and not their actual magnitudes [27]. Therefore, to describe the appearance of a model in terms of its perceptual attributes, a color appearance model can be used. There exist many different colour appearance models, the most widel- used being the sophisticated CIECAM02 model and the more basic CIELAB and CIELUV colour spaces. Given that the CIECAM02 model is very elaborate and is designed to evaluate colour appearance under very specific environmental circumstances, using it is unnecessary for our current LSC window purposes, since environmental factors are not being evaluated [52]. In our case, the CIELUV color space will be used in favor of CIELAB, as the former is optimized for the characterization of self-luminous colour displays and the latter for colored surfaces and dyes.

The difference between a chromaticity space and a color space is that the latter has an extra variable to represent the relative brightness of a color. The three quantities of the CIELUV uniform colour space are given by the following formulas:

(17) $$L^{*}=116f\left(Y/Y_{n}\right)-16,$$

(18) $$u^{*}=13L^{*}\left(u^{\prime }-u_{n}^{\prime }\right),$$

(19) $$v^{*}=13L^{*}\left(v^{\prime }-v_{n}^{\prime }\right),$$

where $f(Y/Y_{n})={\left(Y/Y_{n}\right)}^{1/3}$ for $Y/Y_{n}>{\left(6/29\right)}^{3}$ and $f\left(Y/Y_{n}\right)=\left(841/108\right)\left(Y/Y_{n}\right)+4/29$ for $Y/Y_{n}\leq {\left(6/29\right)}^{3}$. $Y_{n},u_{n}^{\prime },$ and $v_{n}^{\prime }$ represent the values of $Y,u^{\prime },$ and $v^{\prime }$ for a reference white, typically the white point in a $u^{\prime }v^{\prime }$ chromaticity diagram. The $L^{*}$ factor, or the lightness of a color, is the attribute that is missing in the chromaticity diagrams but which can be derived from the uniform color spaces. It can be conceptualized as indicating where a color lies on the gray-scale, from white to black, of that particular chromaticity.

When a test color is exposed to different illuminants, it will produce different chromaticity coordinates. This color shift when quantified as the Euclidean distance between the two colors results in the color difference ΔE:

(20) $$\Delta E=\sqrt{{\left(\Delta L^{*}\right)}^{2}+{\left(\Delta u^{*}\right)}^{2}+{\left(\Delta v^{*}\right)}^{2}}.$$

In our case, the test illuminant is the light transmitted from an LSC window while the reference illuminant is determined by the CCT of the test color. If the CCT value is less than 5000 K, then the ideal blackbody radiator is used as a reference illuminant, otherwise the natural daylight spectrum is used. Then, the special color rendering index is calculated for each sample with the formula:

(21) $$R_{i}=100-\Delta E_{i}.$$

The average of the twelve special coloring indices, for the colors in Table 1, gives the CRI value for the given illuminant.

## 5. Results

### 5.1. Colorimetry Assessment

Light transmittance of the commercial glass used for windows or facades applications varies according to the type of the glass, the wavelength of the incoming radiation, and the thickness of the glass. The overall transmittance in the visible part of the spectrum has values between 80–92% [53], but it can be lower, if required for the utility of the building or architectural design. By embedding luminophore molecules inside the host material, part of the visible spectrum will be absorbed and, therefore, the transmittance will change.

In this study, we simulated LSC plates with dimensions of 10 × 10 × 0.5 cm using the absorption and emission profiles of the luminophores, described in the previous section. The absorption curves were normalized in such a way that the average optical transmittance ($T_{\mathrm{vis}}$) in the visible part (390–700 nm) of the plates varied from 0.9, which is a common value for conventional windows, down to 0.5 for moderately tinted glass. The incident light spectrum ($S_{i}$) was obtained from the AM1.5G spectrum and, for every absorption and emission profile, a total of one million photons were simulated. The photons were emitted normally to the front surface of the LSC window. Photons that passed through the waveguide unabsorbed were collected and are referred to as the transmission spectrum ($S_{t}$). Scattering and absorption losses in the matrix were not taken into account.

An LSC window which is characterized by spectral transmittance T(λ) modifies the SPD of the incoming daylight, $S_{i}$:

(22) $$S_{t}\left(\lambda \right)=S_{i}\left(\lambda \right)T\left(\lambda \right).$$

**Figure 7:**
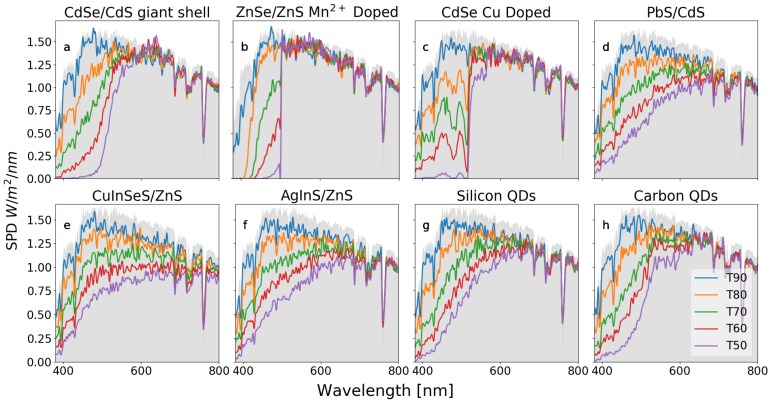
Simulated spectral power distribution of transmitted irradiance for eight different luminophores with five different optical transmittance values. T90 represents a transmittance of 90%, while T50 represents a transmittance of 50%.

As implied by the above equation, the transmitted irradiation $S_{t}$ only depends on the incoming irradiation $S_{i}$ and the spectral transmittance T(λ), which is a characteristic material property of the LSC plate with the embedded luminophore. Figure 7 depicts the transmitted SPD of the LSC windows ($S_{t}$) which use the luminophores described in Section 3, with the shaded area in the background representing the AM1.5G spectrum ($S_{i}$). For each case, five different optical transmittance values ($T_{\mathrm{vis}}$) were simulated, starting from a lightly doped scenario $T_{\mathrm{vis}}=90\%$, down to a heavily doped and tinted LSC plate, with $T_{\mathrm{vis}}=50\%$. The value of $T_{\mathrm{vis}}$ only defines the total amount of light transmitted through a medium, compared to the incident value and, therefore, cannot give any further information regarding the SPD and, consequently, the color quality.

The luminophores chosen absorbed strongly in the UV and blue parts of the visible spectrum, but their absorptions weakened considerably beyond 500 nm (Figure 2), and so the resulting transmission SPDs did not exhibit a gradual reduction, uniformly distributed to every wavelength, compared to the AM1.5G spectrum. For $T_{\mathrm{vis}}=90\%$, all of the SPDs roughly followed the AM1.5G outline, but, as $T_{\mathrm{vis}}$ increased, the shape of the SPD was greatly affected by the shape of the absorption spectrum of each individual QD. The absorption peak of the thick shell CdSe/CdS QDs at 480 nm resulted in a shoulder at the transmission SPD, located at the same wavelength, which became stronger for $T_{\mathrm{vis}}=60\%$ and $T_{\mathrm{vis}}=50\%$. The rapid decrease of the absorption coefficient of the ${\mathrm{Mn}}^{2+}$-doped ZnSe/ZnS QDs beyond 500 nm, and of the CdSe Cu-doped QDs beyond 530 nm, was reflected as a steep decrease in the transmission SPD magnitudes. At this point, it is important to highlight that, in order to achieve the expected $T_{\mathrm{vis}}$ values, the ${\mathrm{Mn}}^{2+}$-doped ZnSe/ZnS, thick shell CdSe/CdS QDs, and CdSe Cu-doped QDs had to totally absorb every photon with wavelength smaller than 500 nm, which resulted in total elimination of the blue and green components of the visible spectrum. On the other hand, the PbS/CdS and CuInSeS/ZnS QDs, with a long absorption tail that expanded to the infrared, achieved the same level of transparency by absorbing photons from the whole range of the visible spectrum.

**Figure 8:**
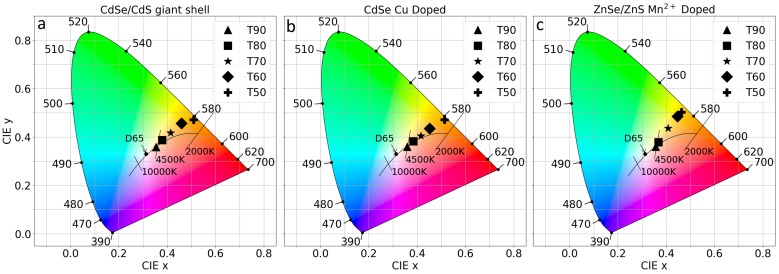
Chromaticity diagram showing data for the D65 illuminant when filtered through an LSC window for (**a**) the thick shell CdSe/CdS, (**b**) the ${\mathrm{Mn}}^{2+}$-doped ZnSe/ZnS, and (**c**) the Cu-doped CdSe QDs.

The actual color of the daylight, as it is transmitted through an LSC window, can be represented as a point on a chromaticity diagram. In the case of the thick shell CdSe/CdS QDs (Figure 8a), the high transmittance scenario (T90) had chromaticity coordinates very close to the average daylight (D65) value. But, as transmittance decreased, the gradual reduction of the violet and blue components from the final SPD increased the chromaticity difference from D65 towards the orange part of the spectrum and away from the Planckian locus. The same pattern was followed by the Cu doped CdSe QDs (Figure 8b), but the limited absorption of ${\mathrm{Mn}}^{2+}$-doped ZnSe/ZnS (Figure 8c), that blocked almost every violet, blue, and green wavelength, resulted in a yellow light. Additionally, the elimination of specific wavelengths resulted in greater chromaticity differences to achieve the same level of transparency and created a stepwise transmission spectrum which had a very different shape, compared to a blackbody radiator, which is depicted by the distance of T60 and T50 points from the Planckian locus.

**Figure 9:**
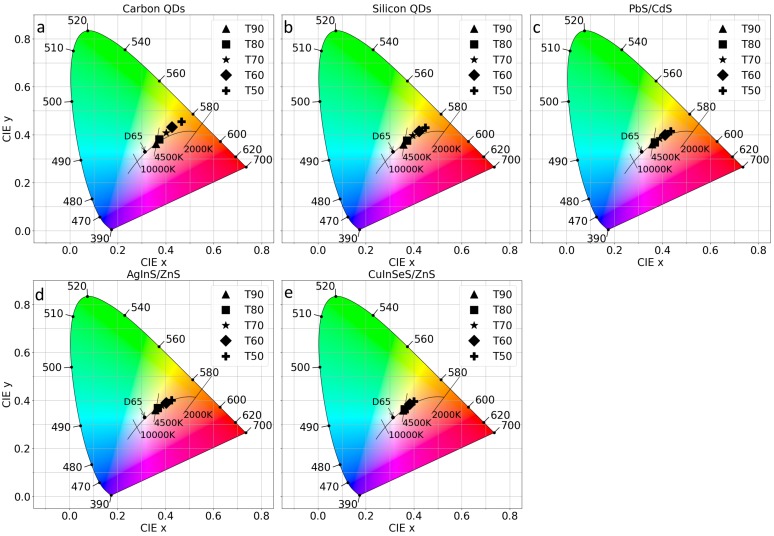
Chromaticity diagram showing data for the D65 illuminant when filtered through an LSC window for (**a**) Carbon, (**b**) Silicon, (**c**) PbS/CdS, (**d**) AgInS/ZnS, and (**e**) CuInSeS/ZnS QDs.

From the chromaticity diagrams in Figure 9, it is clear that, as the absorption spectrum extends to higher wavelengths and it is not limited to a very specific area of the visible spectrum, then the chromaticity differences were reduced, as in the case of Carbon, Silicon, and PbS/CdS QDs (Figure 9a–c). The minimum of chromaticity differences for the same range of transmission values are observed for AgInS/ZnS and CuInSeS/ZnS QDs (Figure 9d,e), where all the points were located in the same area of the chromaticity diagram and very close to the Planckian locus.

The importance of the Planckian locus in color rendering is related to the fact that the majority of visual human experiences are associated with light whose spectrum and color is closely related to blackbody radiation, which is characterized by temperature as a single parameter. As the transmission spectrum is responsible for the color perception of a viewer, the CRI value will be calculated based on that. The first step is to calculate the CCT of the illuminant, which will define the reference illuminant as the basis for the comparison and can be deducted from the chromaticity diagram by projecting the calculated chromaticity coordinates (x,y) on the Planckian locus, using an iso-temperature line.

**Figure 10:**
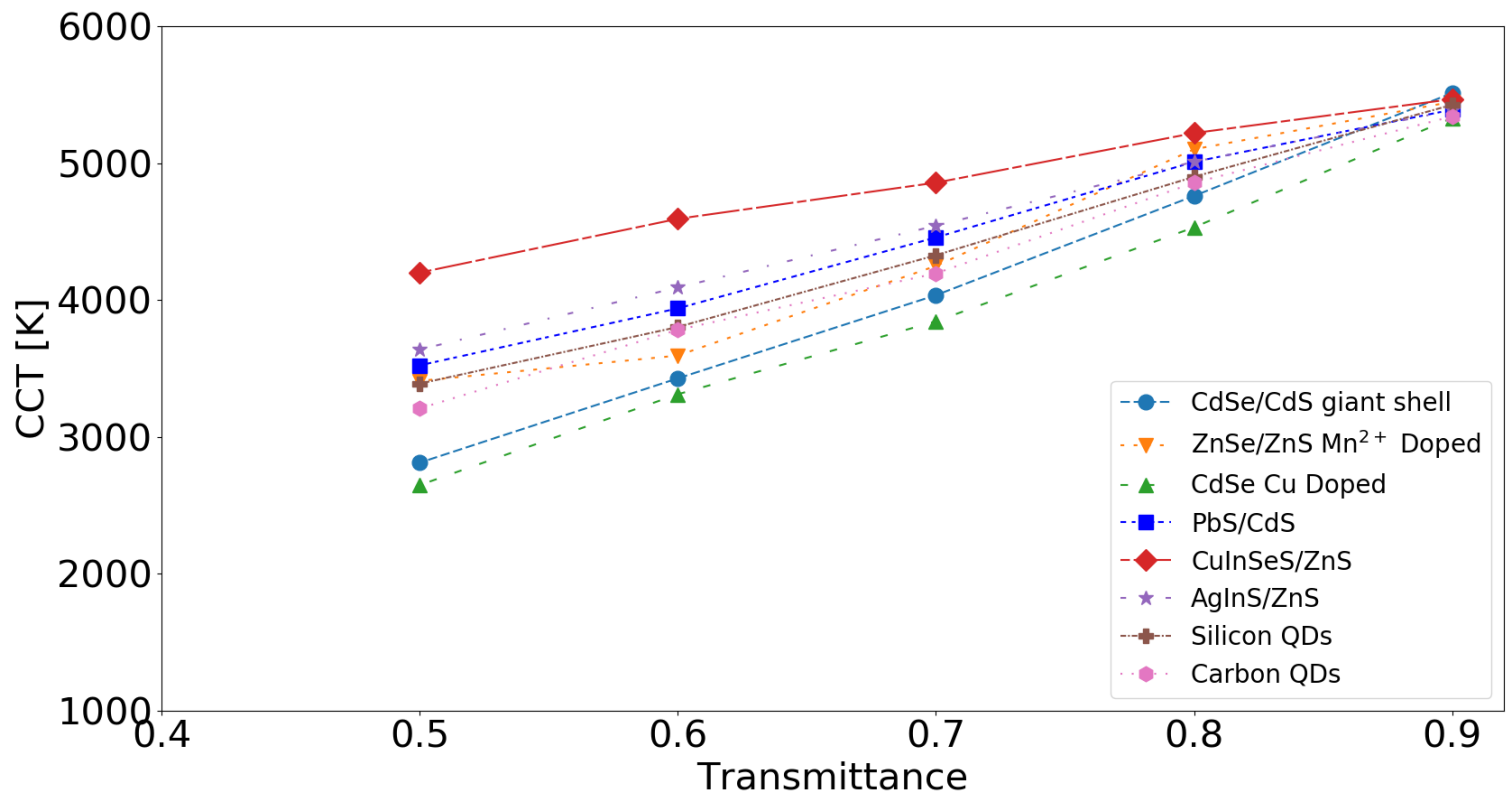
Correlated color temperature (CCT) in Kelvin, plotted against $T_{\mathrm{vis}}$.

The CCT in Kelvin is projected against $T_{\mathrm{vis}}$ in Figure 10. The CCT values for the LSC windows that have $T_{\mathrm{vis}}=90\%$ have a low level of variation, having a lowest value of 5330 K for the Cu-doped CdSe/CdS and a maximum of 5512 K for the giant-shell CdSe/CdS. These values are very close to the CCT of average daylight, which is reasonable, considering the proximity of the T90 points to the D65 point on the chromaticity diagrams. As the concentration of the luminophores in the devices increased, the power density in specific wavelengths decreased, altering the CCT of the resulted SPDs. High CCT values are associated with blue colors and, therefore, the stronger the absorption of the high energy photons, the lower the final CCT value. Consequently, the CCT for $T_{\mathrm{vis}}=50\%$ samples had a lower value than the $T_{\mathrm{vis}}=90\%$ samples, for every luminophore. Moreover, a higher variation was observed for the $T_{\mathrm{vis}}=50\%$ samples, with CuInSeS/ZnS reaching 4200 K and Cu-doped CdSe/CdS 2647 K. Therefore, while in the $T_{\mathrm{vis}}=90\%$ case, the difference between the higher and the lower temperature was 182 K, for $T_{\mathrm{vis}}=50\%$ it was more than eight times higher, at 1553 K.

**Figure 11:**
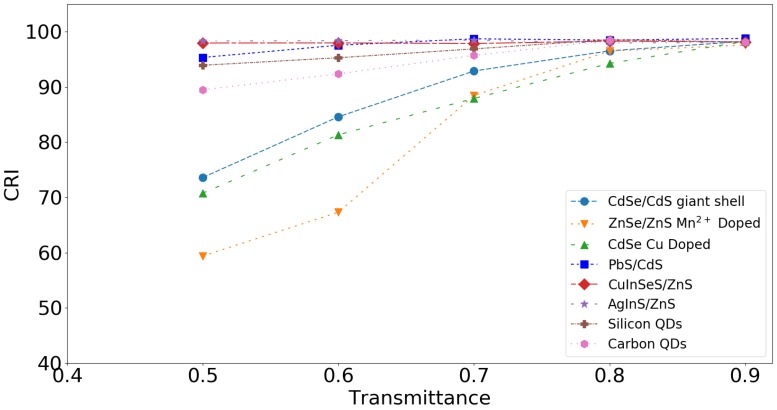
Color rendering index (CRI), plotted against $T_{\mathrm{vis}}$.

Light transmitted through an LSC window so far has been characterized by two indicators. $T_{\mathrm{vis}}$ measures the intensity of the indoor light compared to natural daylight, and CCT measures the amount of power that is radiated indicating the “warmth” of the light in the room. The third indicator, CRI, which is presented in Figure 11, in comparison with $T_{\mathrm{vis}}$, quantifies the effect of the illuminant, accounting for human vision. In Figure 10, it was shown that samples with high $T_{\mathrm{vis}}$ had very similar CCT values, and the same applies for CRI. At $T_{\mathrm{vis}}=90\%$, every sample scored above 97.6, which makes them exceptional for every architectural application. As $T_{\mathrm{vis}}$ decreased, so did the CRI; but with a different rate for every sample. Half of the luminophores, and specifically CuInSeS/ZnS, AgInS/ZnS, PbS/CdS, and Silicon QDs, maintained their remarkable color-rendering properties at high concentrations and, therefore, high CRI values were reported, even at $T_{\mathrm{vis}}=50\%$. The lowest measured CRI value in this case was 94, for the Silicon QDs, and the highest was 98, for CuInSeS/ZnS QDs. Carbon QDs, having a CRI value of 89 for the maximum concentration, still offered a good level of color neutrality and, therefore, can be considered as a promising option. On the contrary, the color rendering performance of the giant shell CdSe/CdS, Cu-doped CdSe, and ${\mathrm{Mn}}^{2+}$-doped ZnSe/ZnS QDs dropped drastically with decreasing $T_{\mathrm{vis}}$. Specifically, for $T_{\mathrm{vis}}<0.7\%$, the light filtered by the aforementioned luminophores offered very limited visual comfort and, therefore, they can not be recommended for a domestic or working environment.

**Figure 12:**
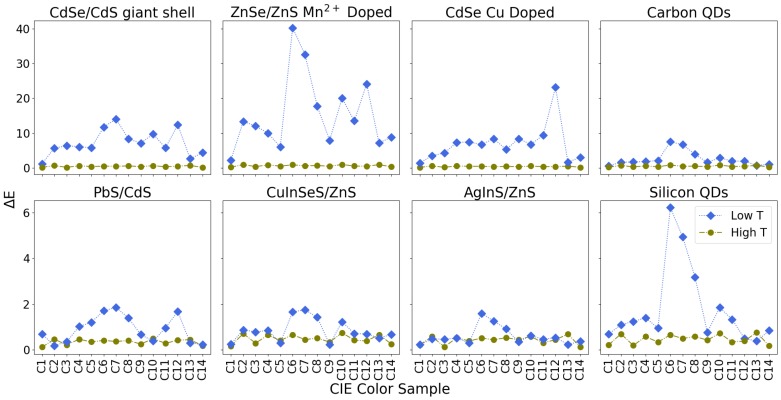
ΔE for the 14 CIE color samples for high ($T_{\mathrm{vis}}=90\%$) and low ($T_{\mathrm{vis}}=50\%$) transmittance values.

The CRI is a single number, describing the overall level of visual comfort provided by the illuminant and, therefore, can not show how the illuminant affects every color individually. As it is practically impossible to examine each and every color, we are going to focus only on the 14 CIE color samples described in Table 1. The color changes (ΔE) of all the test-color samples, achieved between $T_{\mathrm{vis}}=90\%$ and $T_{\mathrm{vis}}=50\%$, of the LSC windows were calculated using Equation (19), and the results are presented in Figure 12. To our knowledge, there is no existing data about acceptable color differences (ΔE) in the same object produced by different illuminants and, therefore, as a matter of comparison, we will present industry standards regarding the largest acceptable difference between two samples, when viewed under the same illuminant. In general, different applications have different tolerances and some colors are more susceptible to a noticeable color difference than others. For automobile tight standards, a pair of coatings may differ only by 1 CIELUV unit (ΔE), in order to be considered as a acceptable match [54]. For computer monitor calibrations, ΔE < 2.5 is considered as the minimal detectable difference for human eyes and a ΔE value between 3 and 6 is acceptable for commercial reproduction [55]. However, these values are the standards in color-sensitive industries and do not represent the levels of visual comfort or well-being in a room with filtered daylight.

As expected, the samples with the lowest CRI at $T_{\mathrm{vis}}=50\%$ exhibited the largest ΔE values. Giant shell CdSe/CdS and Cu-doped CdSe QDs distorted yellows and greens (C2–C5) at a moderate level (ΔE between 3–6) and reds (ΔE between 5–9 for C8–C9), but blues are distorted at a higher degree (ΔE between 11–23 for C6–C7 and C12). On the other hand, ${\mathrm{Mn}}^{2+}$-doped ZnSe/ZnS exhibited high ΔE for most of the samples, indicating a large distortion in every part of the color spectrum—which would be uncomfortable for potential building users under this type of light. Carbon and Silicon QDs exhibited a maximum of color change more in the blue area (C6–C7) with ΔE values between 6 and 7 and less in the purple-red (C8) with ΔE = 4. Light filtered by PbS/CdS, CuInSeS/ZnS, and AgInS/ZnS QDs only caused a slight distortion, mainly in the C6–C8 samples but, since ΔE was below 2 CIELAB units, these values would comply even with the strict industrial standards.

### 5.2. Performance Analysis

**Figure 13:**
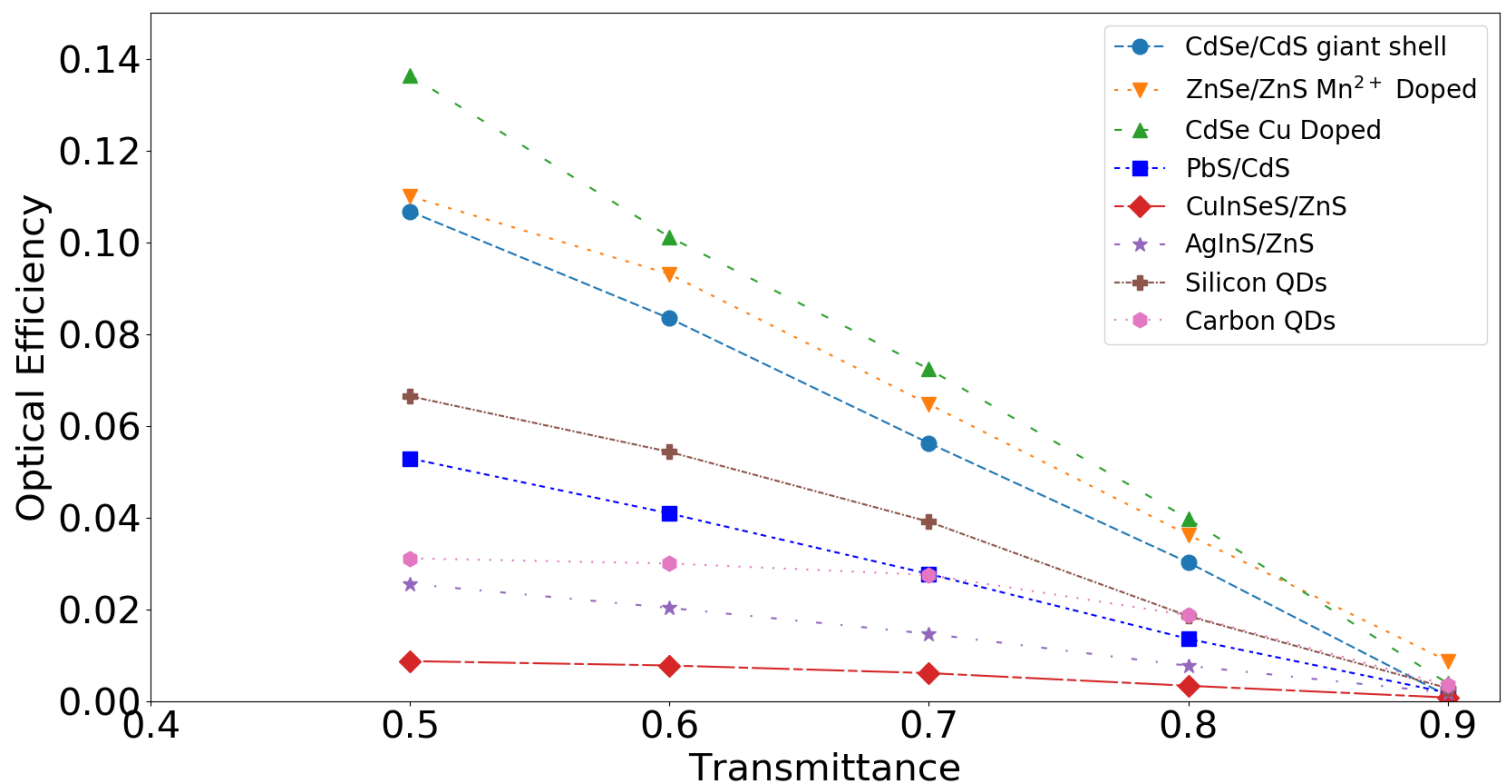
Optical efficiency, plotted against Tvis.

LSC windows primarily operate as light harvesting devices and electrical generators. Therefore, besides the aesthetics and visual comfort that they can provide, the overall performance is probably the most significant parameter. The performance of LSC devices depends on many different aspects, such as the geometry of the plate, the Stokes Shift, and the $\eta_{LQE}$ of the luminophores. In this case, since the dimensions of the simulated LSC windows are the same, then the geometry can be neglected for the comparative analysis. First, the LSC windows will be assessed independently of the solar cells attached to their sides, using the optical efficiency ($\eta_{opt}$), from Equation (5), as the main indicator. In Figure 13, the values of $\eta_{opt}$ are presented, compared to $T_{\mathrm{vis}}$.

For every luminophore, $\eta_{opt}$ increased with decreasing $T_{\mathrm{vis}}$, as more light was captured and redirected to the edges of the device. However, not every luminophore exhibited the same rate of performance increase. The highest rate of increase and the highest $\eta_{opt}=13.6\%$ for $T_{\mathrm{vis}}=50\%$ was observed for the Cu-doped CdSe/CdS QDs, with a low reabsorption spectrum and high $\eta_{LQE}=70\%$, which was also the highest among the examined luminophores. Then, the ${\mathrm{Mn}}^{2+}$-doped ZnSe/ZnS and the Cu-doped CdSe/CdS followed, with $\eta_{opt}=11.2\%$ and $\eta_{opt}=10.7\%$, for $T_{\mathrm{vis}}=50\%$, respectively; both of these had the second and third highest $\eta_{LQE}$ ($\eta_{LQE}=50\%$ and $\eta_{LQE}=45\%$, respectively), as can be seen from Table 1, and both exhibited negligible reabsorption losses. Limited reabsorption losses were also reported for the Silicon QDs but, due to the moderate $\eta_{LQE}=45\%$, $\eta_{opt}$ only reached 6.6%. Despite the broad absorption spectrum and the $\eta_{LQE}=50\%$ of the PbS/CdS QDs, only 5.3% of the incident power was absorbed and successfully waveguided to the edge of the device. Finally, Carbon ($\eta_{LQE}=40\%$), AgInS/ZnS ($\eta_{LQE}=30\%$), and CuInSeS/ZnS ($\eta_{LQE}=40\%$), which suffered from reabsorption losses, only reached $\eta_{opt}=3.1\%$, $\eta_{opt}=2.5\%$, and $\eta_{opt}=0.9\%$, respectively.

**Figure 14:**
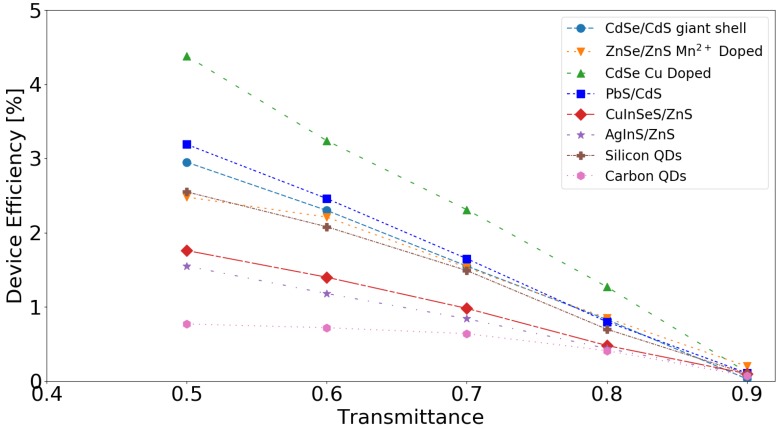
Percentage of device efficiency, plotted against Tvis.

In principle, since the LSC windows had the same $T_{\mathrm{vis}}$, then the ones that absorbed beyond 700 nm to the NIR should have had a slight advantage, as they were able to collect non-visible photons, maintaining the same $T_{\mathrm{vis}}$. However, as was shown in Figure 13, a high $\eta_{LQE}$ and a low level of reabsorption were the more important factors in achieving a high $\eta_{opt}$. Additionally, $\eta_{opt}$ can be misleading as an indicator. As the $L_{edge}$ is compared with the incident power on top of the device $L_{in}$, luminophores that emit high-energy photons had an advantage, compared to the ones that emitted in longer wavelengths. Therefore, $\eta_{opt}$, as an indicator, only focuses on the light-harvesting and transferring abilities of an LSC. An elemental component of an LSC device, however, is the attached solar cell, where solar energy is transformed into useful electricity. As was shown in Figure 4, the EQE of a silicon solar cell peaks after 600 nm and, therefore, every photon emitted below that threshold will only be partially utilized. Assuming that the LSC windows will be equipped with silicon solar cells, as they are the most popular and cost-efficient type of solar cell, the overall device efficiency is presented in Figure 14. The device efficiency is defined as the ratio of the power delivered by the solar cell of the LSC ($L_{SC}$) to the incident power on the top of the device ($L_{in}$). For the calculation of the device efficiency, light scattering was neglected and a perfect connection between the LSC and the solar cell was assumed (i.e., without any losses). All four sides of the LSC were covered with solar cells and no back reflector was used.

As expected, device efficiency values were lower than optical efficiency ones. Moreover, the importance of spectral matching is made clear by observing Figure 14, and especially $\eta_{dev}$ for $T_{\mathrm{vis}}=50\%$. Luminophores with an emission peak below 600 nm, such as ${\mathrm{Mn}}^{2+}$-doped ZnSe/ZnS and Carbon QDs are not a good match for the silicon solar cells. While they were rated as second and sixth among the other luminophores, in terms of $\eta_{opt}$, respectively, they dropped to the fourth and eighth position with $\eta_{dev}=2.48\%$ and $\eta_{dev}=0.77\%$. On the contrary, PbS/CdS and CuInSeS/ZnS QDs, with a broad emission spectrum that expands to the NIR benefited the most, having $\eta_{dev}=3.2\%$ and $\eta_{dev}=1.2\%$, respectively. The best-performing luminophore was Cu-doped CdSe, with $\eta_{dev}=4.38\%$. However, in order to exceed $\eta_{dev}=2\%$, a high concentration of luminophores is necessary, resulting in $T_{\mathrm{vis}}\leq 60\%$, in most cases (PbS/CdS, Cu-doped CdSe, ${\mathrm{Mn}}^{2+}$-doped ZnSe/ZnS, Silicon, and giant-shell CdSe/CdS), while, for others, it is not possible at all (CuInSeS/ZnS, AgInS/ZnS, and Carbon QDs).

## 6. Conclusions

When energy generation is integrated into a building, then some elements have more than one functionality. Nonetheless, any intervention to the building envelope has to be carefully evaluated, having potential to alter the well-being of the occupants. In the case of LSC windows, if visual comfort is compromised in favor of power generation, then they will not become a viable solution. Different types of luminophores are being developed and every one of them is a potential candidate for future applications. However, since their optical properties are greatly affected by their absorption spectrum, they cannot be sufficiently described using $T_{\mathrm{vis}}$, alone. Therefore, we have used CRI and CCT to describe and evaluate different aspects of the filtered light.

Since every luminophore has a high absorption coefficient in the UV and the blue parts of the spectrum, the resulting light will be warmer than the average daylight. Although a flat spectrum provides a rough guide for color neutrality, this is not something that can be easily achieved by a QD. However, we have shown that luminophores that have an extended absorption spectrum can achieve high CRI values (above 90%), even when transmittance is decreased down to 50%. Luminophores having an absorption spectrum close to CuInSeS/ZnS, AgInS/ZnS, or Silicon QDs, therefore, can be considered as potential candidates for architectural applications. Luminophores whose absorption covers the visible spectrum only partially, such as ${\mathrm{Mn}}^{2+}$-doped ZnSe/ZnS, exhibit low colorimetric performance. The narrow absorption spectrum not only creates color distortions but, at the same time, is a hindering factor for optimal coupling with silicon solar cells. The high-energy emission photons that are usually related to these types of luminophores limit the overall efficiency of the device. The results of this study show that only PbS/CdS QDs managed to achieve a relatively high $\eta_{dev}$ while maintaining a CRI above 90.

Enhancing or suppressing certain parts of the transmission spectrum might be an impossible task for a single type of QD. Therefore, different luminophores may need to be used, either in the same waveguide or in a stacked design, consisting of two or more separate plates, using different types of solar cells for maximum efficiency.

## Figures and Tables

**Table 1:** List of Commission Internationale de l’Eclairage (CIE) Test Color Samples.

Code	Munsell Notation	Color Appearance under Daylight	Sample
C1	7.5 R 6/4	Light grayish red	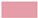
C2	5 Y6/4	Dark grayish yellow	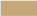
C3	5 GY 6/8	Strong yellow green	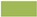
C4	2.5 G 6/6	Moderate yellowish green	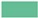
C5	10 BG 6/4	Light bluish green	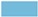
C6	5 PB 6/8	Light blue	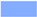
C7	2.5 P 6/8	Light violet	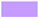
C8	10 P 6/8	Light reddish purple	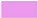
C9	4.5 R 4/13	Strong red	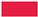
C10	5 Y 8/10	Strong yellow	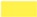
C11	4.5 G 5/8	Strong green	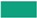
C12	3 PB 3/11	Strong blue	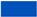
C13	5 YR 8/4	Light yellowish pink	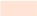
C14	5 GY 4/4	Moderate olive green	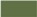

**Table 2:** Emission properties of luminophores.

Luminophore	Emission Peak (nm)	FWHM (nm)	Quantum Yield (%)
CdSe/CdS–giant shell	640	60	45
ZnSe/ZnS ${\mathrm{Mn}}^{2+}$ Doped	590	80	50
CdSe Cu Doped	705	110	70
PbS/CdS	890	160	50
CuInSeS/ZnS	960	180	40
AgInS/ZnS	900	290	30
Silicon QDs	830	120	45
Carbon QDs	550	110	40
